# A promising antitumor activity of evodiamine incorporated in hydroxypropyl-β-cyclodextrin: pro-apoptotic activity in human hepatoma HepG2 cells

**DOI:** 10.1186/s13065-016-0191-y

**Published:** 2016-07-25

**Authors:** Chao Qiu, Li-Na Gao, Kuo Yan, Yuan-Lu Cui, Ye Zhang

**Affiliations:** Research Center of Traditional Chinese Medicine, Tianjin University of Traditional Chinese Medicine, No. 88 YuQuan Road, Nankai District, Tianjin, 300193 China; Tianjin State Key Laboratory of Modern Chinese Medicine, Tianjin University of Traditional Chinese Medicine, Tianjin, 300193 China; Department of Pharmaceutical Sciences, Zibo Vocational Institute, Zibo, 255314 Shandong China

**Keywords:** Evodiamine, Hydroxypropyl-β-cyclodextrin, Apoptosis, Antitumor, P-glycoprotein

## Abstract

**Background:**

Evodiamine has gained wide interests recently because of its antitumor activities. However, a superior bioavailability is required to achieve better efficacy due to its poor water solubility. The aim of this study was to enhance the evodiamine’s aqueous solubility by preparing evodiamine/hydroxypropyl-β-cyclodextrin (EVO/HP-β-CD) inclusion complex, which is incorporated evodiamine into HP-β-CD, and compare the antitumor activities before and after inclusion with HP-β-CD in human hepatoma HepG2 cells.

**Results:**

The EVO/HP-β-CD inclusion complexes were prepared by the kneading method and structurally characterized. P-glycoprotein ATPase assays firstly demonstrated that evodiamine was a substrate of P-glycoprotein, while HP-β-CD and EVO/HP-β-CD inclusion complexes inhibited P-glycoprotein by blocking P-glycoprotein ATPase activity. The EVO/HP-β-CD inclusion complexes may be a promising anticancer drug candidate without drug resistance. After given evodiamine or EVO/HP-β-CD inclusion complexes intervention, cell viability evaluation indicated that the half inhibition concentration of evodiamine and EVO/HP-β-CD inclusion complexes on HepG2 cells was 8.516 and 0.977 μM, respectively. The caspase-3 enzyme activity analysis and Annexin V/PI double-staining revealed that EVO/HP-β-CD inclusion complexes possessed better antitumor activities than evodiamine. Additionally, Hoechst 33258 staining and terminal deoxynucleotidyl transferase-mediated dUTP nick-end labelling assay demonstrated that EVO/HP-β-CD inclusion complexes induced HepG2 cell apoptosis more effectively than evodiamine.

**Conclusions:**

The improved antitumor activities of evodiamine were attributed to the enhanced solubility and P-glycoprotein inhibition by HP-β-CD. These results are promising for the drug administration of EVO/HP-β-CD inclusion complexes to enhance the bioavailability of evodiamine in vivo.

**Electronic supplementary material:**

The online version of this article (doi:10.1186/s13065-016-0191-y) contains supplementary material, which is available to authorized users.

## Background

**Fig. 1:**
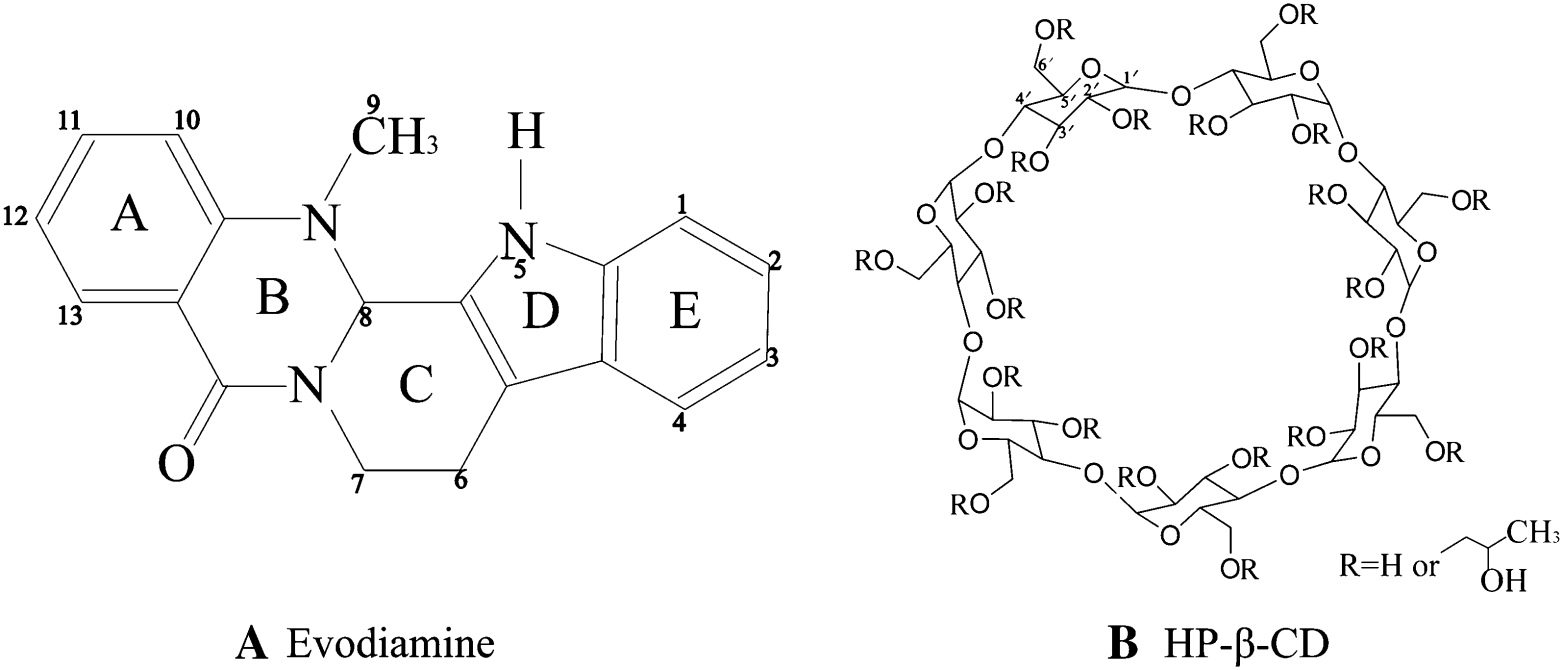
The chemical structures and labelled protons of **A** evodiamine and **B** HP-β-CD

Evodiamine (Fig. 1A), a quinolone alkaloid, is a major bioactive component extracted from the fruit of *Evodiarutaecarpa* (Juss.) Benth. It participates in diverse pharmacological bioactivities, such as antiobesity [1, 2], anti-cancer [3–5], and analgesic activities [6]. It plays a role in regulating the expression of the serotonin transporter [7], improving cognitive abilities in transgenic mouse models of Alzheimer’s disease [8], and stimulating catecholamine secretion [9]. However, its poor water solubility hampers its application in pharmaceutical fields. Therefore, it is necessary to increase its aqueous solubility to improve its pharmaceutical properties. In recent years, several studies have been devoted to the development of novel evodiamine formulations to improve its bioavailability, including phospholipid complexes [10] and microemulsions [11, 12].

Cyclodextrins (CDs), which are cyclic oligosaccharides in the shape of truncated cones, fascinate many scientists because of their hydrophilic outer surfaces and somewhat hydrophobic central cavity. CDs can form water-soluble inclusion complexes with various poorly water-soluble compounds, thereby enhancing the bioavailability of insoluble drugs by increasing the drug solubility, dissolution, and/or drug permeability [13–15]. It has been reported that CDs or their chemically modified derivatives can improve water solubility and the bioavailability of drugs with poor water solubilities [16, 17]. The most commonly used CDs are β-cyclodextrin (β-CD) and its chemically modified derivatives, such as hydroxypropyl-β-cyclodextrin (HP-β-CD) and sulfobutylether-β-CD. HP-β-CD has been widely acknowledged for its increased water-solubility, lower toxicity, and higher inclusion ability relative to natural β-CD. It is also known that cancer drug resistance develops because of the presence of multidrug resistance (MDR) genes in cancer cell membranes [18–20]. P-glycoprotein (Pgp), encoded by MDR1 which is the first human ATP-binding cassette (ABC) transporter gene, influences drug absorption, distribution, and secretion. Consequently, it is very important to identify a Pgp inhibitor with few side effects to improve drug absorption in cancer treatments. It has been reported that HP-β-CD inhibits Pgp by blocking Pgp ATPase activity, which could enhance drug absorption [11, 21].

At present, no aqueous formulation of evodiamine is available. In this investigation, evodiamine inclusion complexes were prepared with HP-β-CD to improve the water solubility of evodiamine. Then, the antitumor activities of EVO/HP-β-CD and evodiamine were comparatively studied by measuring caspase activities, Hoechst staining, and TUNEL assays. A Pgp ATPase assay was also used to identify whether HP-β-CD acted as a Pgp inhibitor and whether evodiamine was a Pgp substrate.

## Methods

### Materials

Evodiamine (purity >98 %) was purchased from Nanjing Goren Bio-Technology Co., Ltd. (Nanjing, China). HP-β-CD (CAVASOL^®^HP, molecular mass 1540) was purchased from Wacker Chemie AG (Munich, Germany). 3-(4, 5- dimethylthiazol-2-yl)-2, 5-diphenyltetrazolium bromide (MTT) and Dulbecco’s modified Eagle’s medium (DMEM, high glucose) were purchased from Sigma-Aldrich (St Louis, MO, USA). Double distilled water was obtained from a Milli-Q water purification system (Merck Millipore, Bedford, MA, USA). The Annexin V apoptosis detection kit (FITC) was purchased from eBioscience (San Diego, CA, USA). Bisbenzimide H 33258 (Hoechst 33258) was obtained from Sigma-Aldrich (St Louis, MO, USA).

### Cell culture and drug treatments

The human hepatoblastoma cell line HepG2 was purchased from the Cell Culture Center of the Chinese Academy of Sciences (Shanghai, China) and cultured in DMEM with 10 % foetal bovine serum at 37 °C in a 5 % CO_2_ humidity incubator for 24 h. Then, varying concentrations of evodiamine or equimolar concentrations of EVO/HP-β-CD or HP-β-CD were added to the culture plates. Cells at 80–90 % confluence were used for all of the experiments.

### Phase solubility studies

Phase solubility studies were adapted from a method introduced by Higuchi and Connors et al. [22]. An excess amount of evodiamine (10 mg) was added to 3 mL deionized water with various concentrations of HP-β-CD (0–400 mM) in 20 mL glass vials. The suspension solution was agitated on an IKA^®^ RO10 magnetic stirrer (IKA, Germany) at 800 rpm and room temperature (25 °C) for 24 h and then filtered through a 0.45 μm pore size PES syringe filter (Millipore, USA) to obtain a clear solution, which was then assayed by high-performance liquid chromatography (HPLC). The assay was performed on a Waters 515 HPLC pump and a Waters 2695 system equipped with a Waters 2487 dual wavelength absorbance detector at 225 nm. Ten microliters of sample was injected into a Kromasil C18 column (4.6 × 250 mm, Eka Chemicals AB, Sweden). The mobile phase consisted of acetonitrile/water (70:30, v/v) containing 0.05 % (v/v) triethylamine and 0.1 % (v/v) phosphoric acid at a flow rate of 1.0 mL/min. All of the test groups were performed in triplicate. The phase solubility curve was obtained by plotting the concentration of the dissolved drug versus the concentration of HP-β-CD.

### Preparation of evodiamine inclusion complexes with HP-β-CD

The inclusion complexes were prepared by the kneading method combined with a stirring method at a molar ratio of 2:1 (HP-β-CD:evodiamine). HP-β-CD was accurately weighed and transferred to a mortar. Subsequently, a small amount of water was added, and the mortar contents were kneaded until a homogeneous paste was obtained. Then, an appropriate amount of evodiamine dispersed in a small amount of ethanol/tetrahydrofuran (2:1, v/v) was added to this paste, and the mixture was continuously kneaded for 45 min at room temperature. Next, the paste was transferred into a round flask containing 50 mL of ethanol. The suspension was stirred at 800 rpm for 24 h at 45 °C. The solution was then filtered with a 0.45 μm pore size nylon membrane syringe filter (Millipore, USA). The solution supernatant was recovered and concentrated, and then the residue was dried in an oven at 45 °C.

### Physicochemical characterization of the samples

#### Differential scanning calorimetry (DSC)

Thermodynamic analyses were performed on a DSC7 differential scanning calorimeter (Perkin-Elmer, Norwalk, CT, USA). Evodiamine, the physical mixture (HP-β-CD: evodiamine = 2:1, mole ratio), EVO/HP-β-CD complexes, and HP-β-CD were accurately weighed (10 mg) and then placed into aluminium pans. The pans were sealed and heated from 30 to 300 °C at a scanning rate of 10 °C/min under a nitrogen flow of 20 mL/min with a blank aluminium pan as a reference.

#### Fourier transform infrared spectroscopy (FTIR)

FTIR spectra of evodiamine, the physical mixture, EVO/HP-β-CD complexes, and HP-β-CD were recorded on a spectrophotometer (ALPHA, Bruker, Germany) in the wave number range of 4000–400/cm using KBr tablets.

#### X-ray powder diffractometry (XRD)

Powder X-ray diffraction patterns of evodiamine, the physical mixture, EVO/HP-β-CD complexes, and HP-β-CD were recorded on a Rigaku D/MAX 2500 V diffractometer (Rigaku, Japan) with Ni-filtered Cu Ka radiation at a voltage of 40 kV and 100 mA current. The scanning rate was 8°/min over a 2θ range from 3 to 50°.

#### Ultraviolet spectroscopy (UV) absorption of evodiamine and its complexes with HP-β-CD

Evodiamine ethanol solutions (final concentration of 2.95 × 10^−4^ M) were added to different concentrations of HP-β-CD (0, 5, 10 and 20 %, w/v) and the combined solutions were treated with ultrasonic cleaner for 30 min at 298 K. Then, the effects of the HP-β-CD on the UV spectra of evodiamine were determined using a DU800 visible–UV spectrophotometer (Beckman Coulter, USA) scanning from 200 to 600 nm.

#### NMR spectra and 2D ^1^H–^1^H rotating frame overhauser effect spectroscopy (2D ROESY)

^1^HNMR spectra for HP-β-CD, evodiamine, and EVO/HP-β-CD complexes were recorded by an INOVA-500 spectrometer (Varian, USA) at 298 K in denudated DMSO. A ROESY experiment was performed on a DRX500 spectrometer (Bruker Biospin, Rheinstetten, Germany) at 298 K in denudated DMSO. Samples were equilibrated for 24 h before measurement.

### Determination of Pgp ATPase activity

The Pgp-Glo™ Assay Systems were used to verify whether EVO/HP-β-CD complexes or HP-β-CD could inhibit Pgp ATPase activity. Briefly, sodium orthovanadate (Na_3_VO_4_), verapamil, or test compounds diluted in assay buffer were added to a 96-well white opaque plate, while the assay buffer was used as negative control. Then, 20 µL of diluted Pgp membranes and 10 µL of 25 mM MgATP were also added to the wells. The plate was incubated at 37 °C for 40 min. Afterwards, the ATP remaining in the wells was detected as a luciferase-generated luminescent signal. Basal Pgp ATPase activity was determined as the difference between the ATP hydrolysis of the negative control and that of the Na_3_VO_4_ sample. Verapamil, a typical Pgp substrate, also served as a positive control.

### Cell viability assay

HepG2 cells were cultured in 96-well plates at a density of 1 × 10^4^ cells/well and incubated in a 37 °C, 5 % CO_2_ incubator for 24 h. Then, the cells were treated with various concentrations of the drugs and incubated for another 24 h. Ten microliters of MTT solution (final concentration of 0.5 mg/mL) was added to the culture medium, and the plates were incubated for 4 h at 37 °C. After aspirating the supernatants, 100 μL of DMSO was added to dissolve the formazan salts, and the absorbance was measured at 490 nm on a Victor X5 plate reader (Perkin Elmer, USA).

### Caspase assays

Cells were seeded at a density of 3 × 10^6^ cells/well in 6-well plates for 24 h and treated with various concentrations of evodiamine, EVO/HP-β-CD complexes, or HP-β-CD for an additional 24 h. Then, the cells were harvested by trypsinization and centrifugation. After washing the cells twice with pre-cooled PBS (pH 7.4), ice-cold cell lysis buffer was added to the cells, which were then placed in an ice bath for 15 min to extract the protein. The caspase-3 and -8 activities were detected with caspase colorimetric assay kits (Enzo Life Sciences, USA). Briefly, 20 µL of cell lysates, 70 µL of assay buffer, and 10 µL of the Ac-DEVD-pNA or Ac-IETD-pNA substrates were added to 96-well plates, mixed, and incubated at 37 °C overnight. After incubation, the absorbance was quantified at 405 nm on a Victor X5 plate reader.

### Hoechst staining

Hoechst 33258 staining is usually used to analyse cell nuclear morphologies after apoptosis. The treated cells (as described in “Caspase assays” section) were fixed with 4 % paraformaldehyde for 10 min in situ and then stained with Hoechst 33258 (5 μg/mL) for 3 min at room temperature in the dark. After washing twice with PBS, the cells were observed on a LSM 710 laser scanning confocal microscope (Carl Zeiss, Germany).

### TUNEL assay

The TUNEL assay was used to confirm apoptosis induced by evodiamine, EVO/HP-β-CD complexes, and HP-β-CD according to the protocol of a commercial kit (Beyotime, China). Cells seeded in 6-well plates were fixed with 4 % paraformaldehyde for 30 min. After washing with PBS, the cells were permeabilized with 0.1 % Triton X-100 in an ice bath for 2 min. Next, the cells were treated with detection buffer, which included fluorescein-12-dUTP and DNA-terminal deoxynucleotidyl transferase, at 37 °C in a humid atmosphere in the dark for 1 h. Finally, the cells were observed and photos were taken with an ECLIPSE-Ti inverted fluorescence microscope (Nikon, Japan).

### Flow cytometry analysis

Cells were seeded in 6-well plates at a density of 9 × 10^5^ cells/well and cultured at 37 °C for 24 h. Then, the cells were treated with 1 µM evodiamine or EVO/HP-β-CD complexes for 26 or 28 h, respectively. The cells were then harvested with 0.25 % trypsin solution (w/v) and washed twice with pre-cooled PBS. Finally, the collected cells were treated according to the protocols of the Annexin V apoptosis detection kit (FITC) and analysed using an Accuri C6 flow cytometer (BD, USA).

### Statistical analysis

One-way analysis of variance (one-way ANOVA) was used to test for significant differences using Origin 8.0 software (MicroCal, USA). *P* values less than 0.05 were considered to be statistically significant. All of the results were expressed as the mean ± the standard deviation (SD).

## Results and discussion

### Results of phase solubility studies

**Fig. 2:**
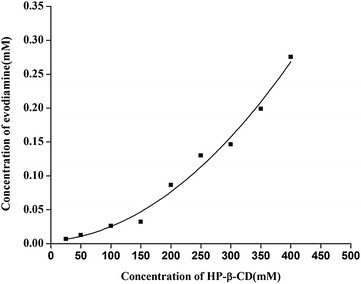
Phase solubility diagram for evodiamine with HP-β-CD

Phase-solubility analysis is a traditional method for determining the influence of inclusion complex agents on a solubilized drug. The phase solubilities of evodiamine and HP-β-CD are shown in Fig. 2, and the curve is identified as A_P_-type (r^2^ = 0.9829), which is increasing positively. According to Uekama et al. [23] this finding indicates that more than one HP-β-CD molecule complexed with one guest molecule at higher HP-β-CD concentrations. As previously reported, drug/cyclodextrin complexes form aggregates or micelles in aqueous solutions and can further solubilize the drug through non-inclusion complexation [24, 25]. The solubility of evodiamine in water increased to 0.2 mmol/L (relative to 60.31 μg/mL) when the concentration of HP-β-CD was 350 mmol/L, which is approximately 158-fold higher than the intrinsic solubility in water (0.38 μg/mL, determined in this study).

### DSC thermograms

**Fig. 3:**
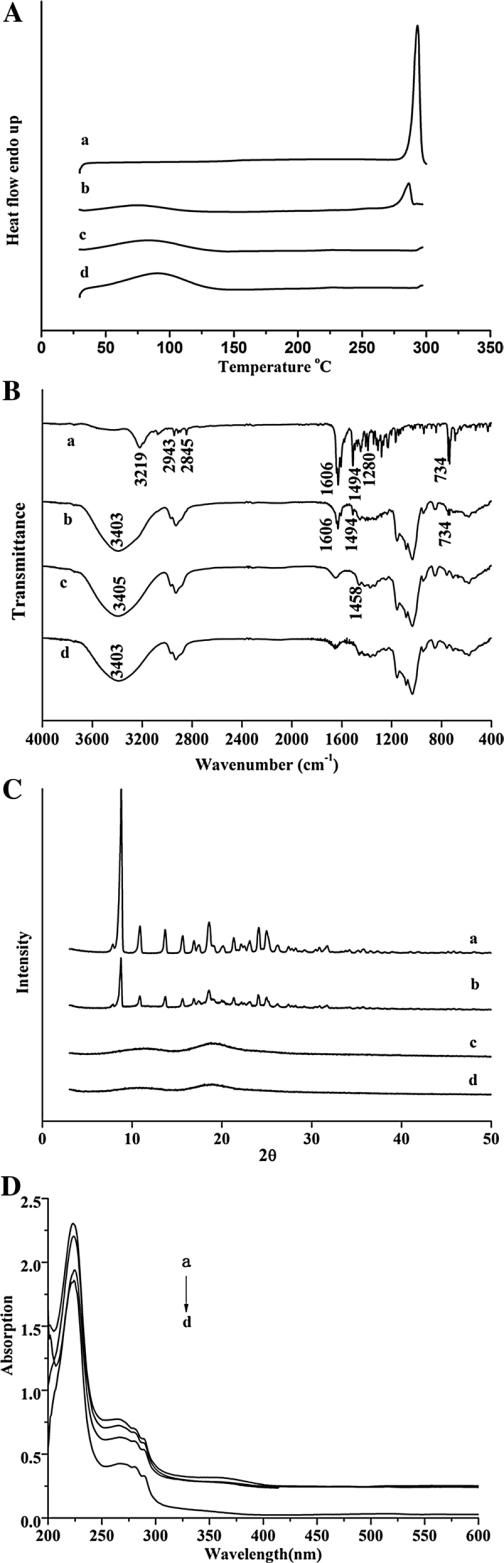
Spectra analysis. **A** DSC curves, **B** FTIR spectra, and **C** XRD patterns of *a* evodiamine, *b* the physical mixture of evodiamine and HP-β-CD, *c* EVO/HP-β-CD complexes, and *d* HP-β-CD. **D** UV spectra of evodiamine: (*a*) in the absence of HP-β-CD, (*b*) in 5 % HP-β-CD, (*c*) in 10 % HP-β-CD, and (*d*) in 20 % HP-β-CD water solution

The thermograms of evodiamine, the physical mixture of evodiamine and HP-β-CD, EVO/HP-β-CD complexes, and HP-β-CD are shown in Fig. 3A. The DSC curve given in Fig. 3A (a) shows a sharp endothermic peak for evodiamine at 293.15 °C (Δ*H* = 128.75 J/g), which is the melting point of evodiamine. The HP-β-CD thermogram shown in Fig. 3A (d) exhibits a very broad endothermic peak near 80 °C due to the loss of water molecules from the cyclodextrin cavity at increasing temperatures. The physical mixture presented endothermic peaks of both evodiamine and HP-β-CD, as shown in Fig. 3A (b), but the melting point of evodiamine was shifted to a lower temperature (286.19 °C) due to interactions between evodiamine and HP-β-CD during the DSC scan. In contrast, the fusion peak of EVO/HP-β-CD did not exhibit the evodiamine endothermic peak, suggesting that the evodiamine crystal characteristic was lost and evodiamine inclusion complexes with HP-β-CD were formed.

### FTIR spectra

The FTIR spectra of evodiamine, the physical mixture of evodiamine and HP-β-CD, EVO/HP-β-CD complexes, and HP-β-CD are shown in Fig. 3B. The evodiamine FTIR spectrum revealed intense absorption at 3219/cm, which is characteristic of –NH group absorption peaks. It also included cyclobenzene absorption peaks, which were observed at 1494, 1280 and 734/cm. The characteristic peaks at 2943 and 2845/cm were attributed to –CH_3_ and –CH_2_ groups, respectively. Another characteristic absorption peak at 1606/cm was for the carbonyl group. The strong absorption peak at 3403/cm is a characteristic peak of the HP-β-CD hydroxyl group. The physical mixture spectrum had peaks that corresponded to both HP-β-CD and evodiamine (3403/cm for the hydroxyl group and 1606/cm for the carbonyl group) but no –NH group or cyclobenzene fingerprint peaks, which may be due to the formation of aggregates during kneading and tableting. Furthermore, the EVO/HP-β-CD FTIR spectrum showed characteristic peaks similar to those of HP-β-CD, which indicates that some of the evodiamine groups might be in the HP-β-CD cavity, suggesting formation of the inclusion complexes.

### XRD

XRD is another effective method for confirming the presence of drug/HP-β-CD inclusion complexes in powder or microcrystalline states. As shown in Fig. 3C, the free evodiamine powder XRD diffraction pattern exhibited sharp peaks, which is characteristic of the pure compound’s crystalline nature, while the XRD diffraction patterns of HP-β-CD confirmed its amorphous state. However, some weaker intensity evodiamine characteristic peaks were still detectable in the physical mixture, as shown in Fig. 3C (b). The EVO/HP-β-CD inclusion complex diffractogram was similar to that of amorphous HP-β-CD; however, no characteristic evodiamine peak was found. The diffractogram of the inclusion complex not showing the crystalline states indicated the formation of the EVO/HP-β-CD inclusion complexes.

### UV spectra

UV spectra can provide powerful evidence of the formation of EVO/HP-β-CD complexes. Therefore, UV spectra were used to confirm evodiamine complex formation with varying HP-β-CD concentrations in aqueous solution. As shown in Fig. 3D, evodiamine ethanol solution alone displayed the strongest absorption peak at 225 nm. With increasing HP-β-CD concentrations, the absorption intensity at 225 nm became weaker. These changes are consistent with some evodiamine chromophore groups entering the HP-β-CD cavity [17, 26].

## ^1^H NMR and 2D ROESY NMR

**Fig. 4:**
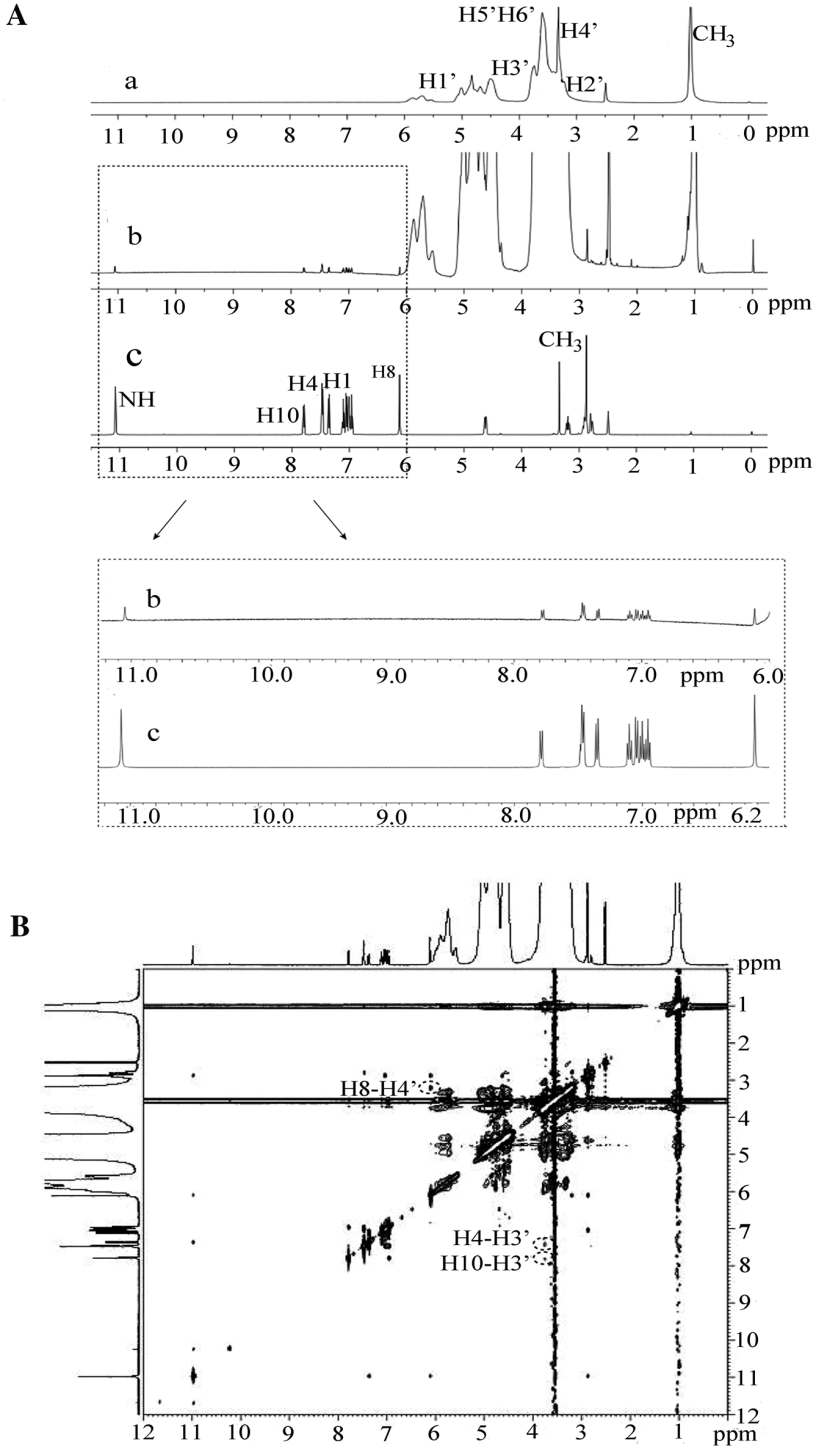
**A** ^1^H NMR spectra. *a* HP-β-CD, *b* EVO/HP-β-CD inclusion complexes, and *c* evodiamine in DMSO-d_6_ at 298 K. **B** ROESY spectrum of the EVO/HP-β-CD inclusion complexes in DMSO-d_6_

^1^H NMR was used to identify the formation of evodiamine inclusion complexes with HP-β-CD. DMSO-d_6_ was chosen as the solvent because the reactive hydrogens (-NH) contained in EVO/HP-β-CD could be substituted by the deuterium atom of D_2_O and to accommodate solubility constraints. DMSO-d_6_ was also chosen as the solvent for analysing the structure of EVO/HP-β-CD, which was soluble in water. The evodiamine ^1^H resonances (Fig. 4A) were assigned as follows: ^1^H NMR (500 MHz, DMSO-d_6_) δ11.07 (s, H-5), 7.79 (d, J = 7.5 Hz, H-10), 7.47 (t, J = 6.5 Hz, H-4), 7.36 (d, J = 8.1 Hz, H-1), 7.10 (t, J = 7.5 Hz, H-11), 7.05 (d, J = 8.2 Hz, H-13), 7.00 (t, J = 7.4 Hz, H-12), 6.96 (t, J = 7.5 Hz, H-2), 6.12 (s, H-8), 2.87 (S, -CH_3_), 4.61, 3.34 (m, H-7), 3.19, and 2.84–2.72 (m, H-6). The formation of EVO/HP-β-CD inclusion complexes was confirmed by shifts in some of the evodiamine proton resonances. As shown in Additional file 1: Table S1, evodiamine signals showed differential shifts from 2 × 10^−2^ to 10^−2^ ppm. However, the combination of protons was different. Namely, the inclusion of evodiamine with HP-β-CD was probably formed by a weak molecular assembly, rather than chemical bonding.

The presence of nuclear over-hauser effect (NOE) cross-peaks between protons from two species indicates spatial contacts within 0.4 nm [27]. A 2D ROESY NMR study was performed to obtain the more conformational information for the EVO/HP-β-CD inclusion complexes. Crosspeaks were observed between the H-4 and H-10 protons on the evodiamine benzene rings and the HP-β-CD H-3′ proton, as well as between the evodiamine H-8 proton and the HP-β-CD H-4′ proton. The simultaneous interaction with both internal and external HP-β-CD protons may be related to the polymeric structure of HP-β-CD. Combined with the ^1^H NMR results above mentioned, all these results indicated that at least two moles of HP-β-CD interacted with one mole of evodiamine (the evodiamine A ring was in one HP-β-CD cavity and the E ring was in another HP-β-CD cavity, as shown in Additional file 2: Figure S1).

### Influence of evodiamine and EVO/HP-β-CD complexes on Pgp ATPase activity

**Fig. 5:**
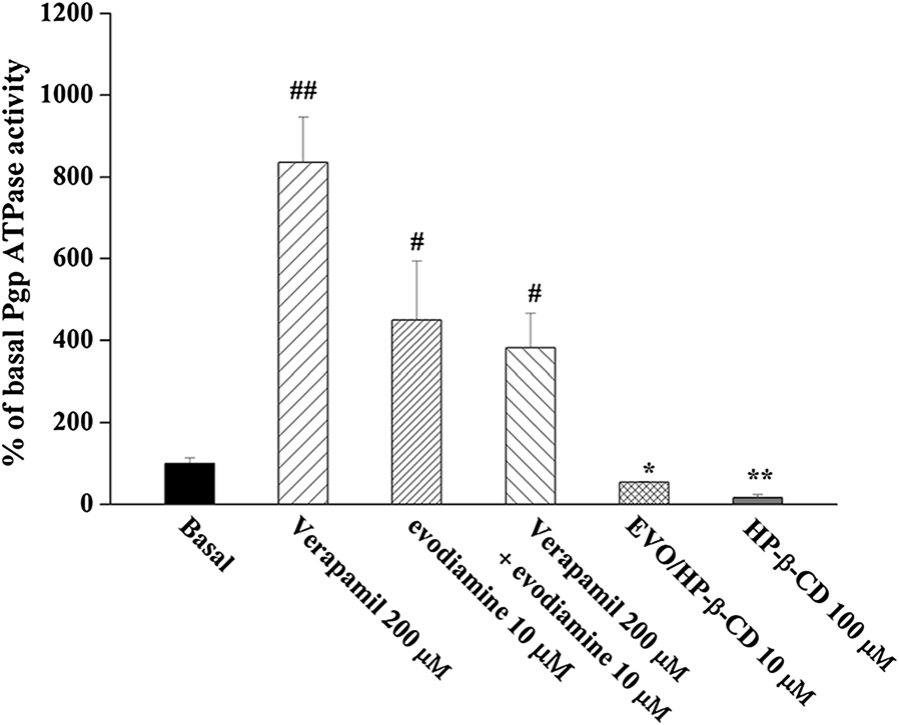
Effects of evodiamine, EVO/HP-β-CD complexes, and HP-β-CD on Pgp ATPase activity. Values given are the mean ± SD (n = 3). ^#^ *P* < 0.05 and ^##^ *P* < 0.01 significantly stimulated basal Pgp ATPase activity. *P < 0.05 and **P < 0.01 significantly inhibited basal Pgp ATPase activity

As shown in Fig. 5, evodiamine alone stimulates basal Pgp ATPase activity and inhibits verapamil-stimulated Pgp ATPase activity, which indicates that evodiamine is a Pgp substrate and binds to the Pgp drug-binding site competitively. HP-β-CD and the EVO/HP-β-CD inclusion complexes significantly inhibited Pgp ATPase activity. Therefore, HP-β-CD could be used to not only improve the solubility of the anticancer drugs but also reduce drug resistance.

### Inhibition of HepG2 cell proliferation by evodiamine and EVO/HP-β-CD complexes

**Fig. 6:**
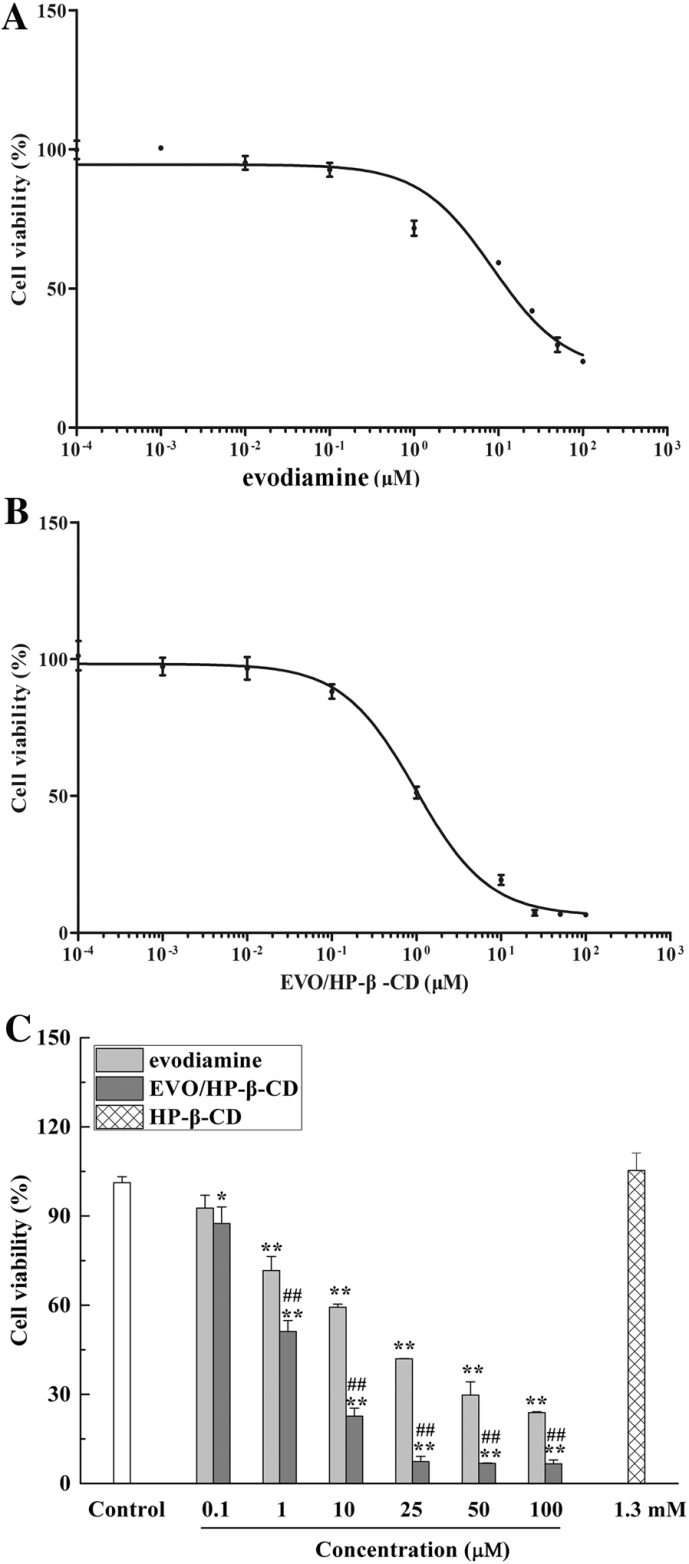
Effects of evodiamine, EVO/HP-β-CD complexes, and HP-β-CD on HepG2 cell proliferation. **A**, **B** The non-linear regression analyses were performed to estimate the half-maximal inhibitory concentrations (IC_50_) of EVO/HP-β-CD and evodiamine. **C** Cell proliferation inhibition of presented concentrations of evodiamine, EVO/HP-β-CD (0.1–100 μM) and HP-β-CD (1.3 mM) was evaluated by MTT. Values given are the mean ± SD (n = 6). ^**^indicates significant differences compared with the control group (*P* < 0.01). ^##^indicates significant differences between evodiamine and EVO/HP-β-CD complexes groups (*P* < 0.01)

MTT assay was used to investigate the effects of evodiamine, HP-β-CD and EVO/HP-β-CD on HepG2 cells proliferation inhibition (Fig. 6). The IC_50_ value of evodiamine and EVO/HP-β-CD on HepG2 cells was 8.516 and 0.977 μM, respectively. To evaluate the anti-cancer effect, we chose 10, 1 and 0.1 μM for further study. Correspondingly, the highest concentration of HP-β-CD was 1.3 mM. Results showed that evodiamine and EVO/HP-β-CD complexes significantly inhibited HepG2 cell viability in a concentration-dependent manner (*P* < 0.01). However, 1.3 mM HP-β-CD had no effect on cell viability. Moreover, the inhibition of EVO/HP-β-CD complexes on HepG2 cells proliferation inhibition was better than that of free evodiamine, suggesting that HP-β-CD improves the cellular uptake efficacy [28].

### Evodiamine and EVO/HP-β-CD complexes induce apoptosis by activating caspase-3 in HepG2 cells

**Fig. 7:**
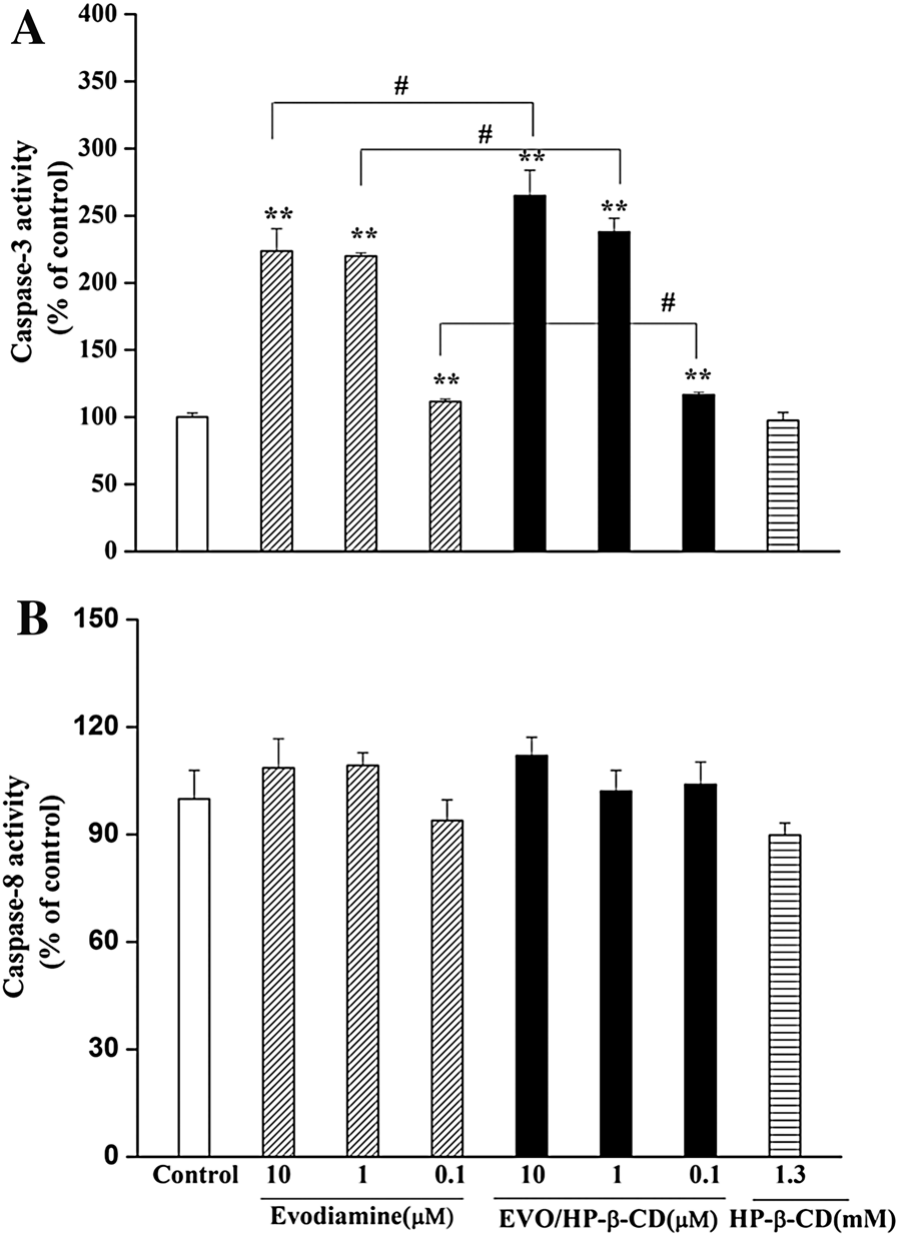
Effects of evodiamine, EVO/HP-β-CD complexes and HP-β-CD on caspases in HepG2 cells. Values of **A** caspase-3 and **B** caspase-8 activities are reported as the mean ± SD (n=3). *Compared with control group, *P* < 0.05. **Compared with control group, *P* < 0.01. ^#^Compared between evodiamine and EVO/HP-β-CD complexes groups, *P* < 0.05

Apoptotic events are closely related to caspase activity, and caspase-3, which is located downstream of the caspase cascade, plays a predominant role in activating effect or caspases that lead to apoptosis. Therefore, caspase-3 and -8 activities were determined by colorimetric analyses. As shown in Fig. 7A, caspase-3 activities in HepG2 cells were enhanced in a concentration-dependent manner after the cells were exposed to either evodiamine or EVO/HP-β-CD. Moreover, the EVO/HP-β-CD complexes could more effectively induce caspase-3 activity than equimolar concentrations of evodiamine alone. In contrast, HP-β-CD alone had no effect on caspase-3 activity. It has been reported that evodiamine is a Pgp substrate; in contrast, HP-β-CD has been shown to be a Pgp inhibitor, suggesting that it could improve the absorption of Pgp substrates. Thus, the inclusion effect of HP-β-CD to enhance evodiamine solubility and inhibit Pgp could improve evodiamine cellular uptake. However, caspase-8 activities were not significantly altered by the same treatments (Fig. 7B). Therefore, caspase-3 is activated by evodiamine and EVO/HP-β-CD complexes to execute apoptosis, implying that caspase-3 is related to evodiamine and EVO/HP-β-CD complex-induced apoptosis.

### HepG2 cell apoptosis induced by evodiamine and EVO/HP-β-CD complexes

**Fig. 8:**
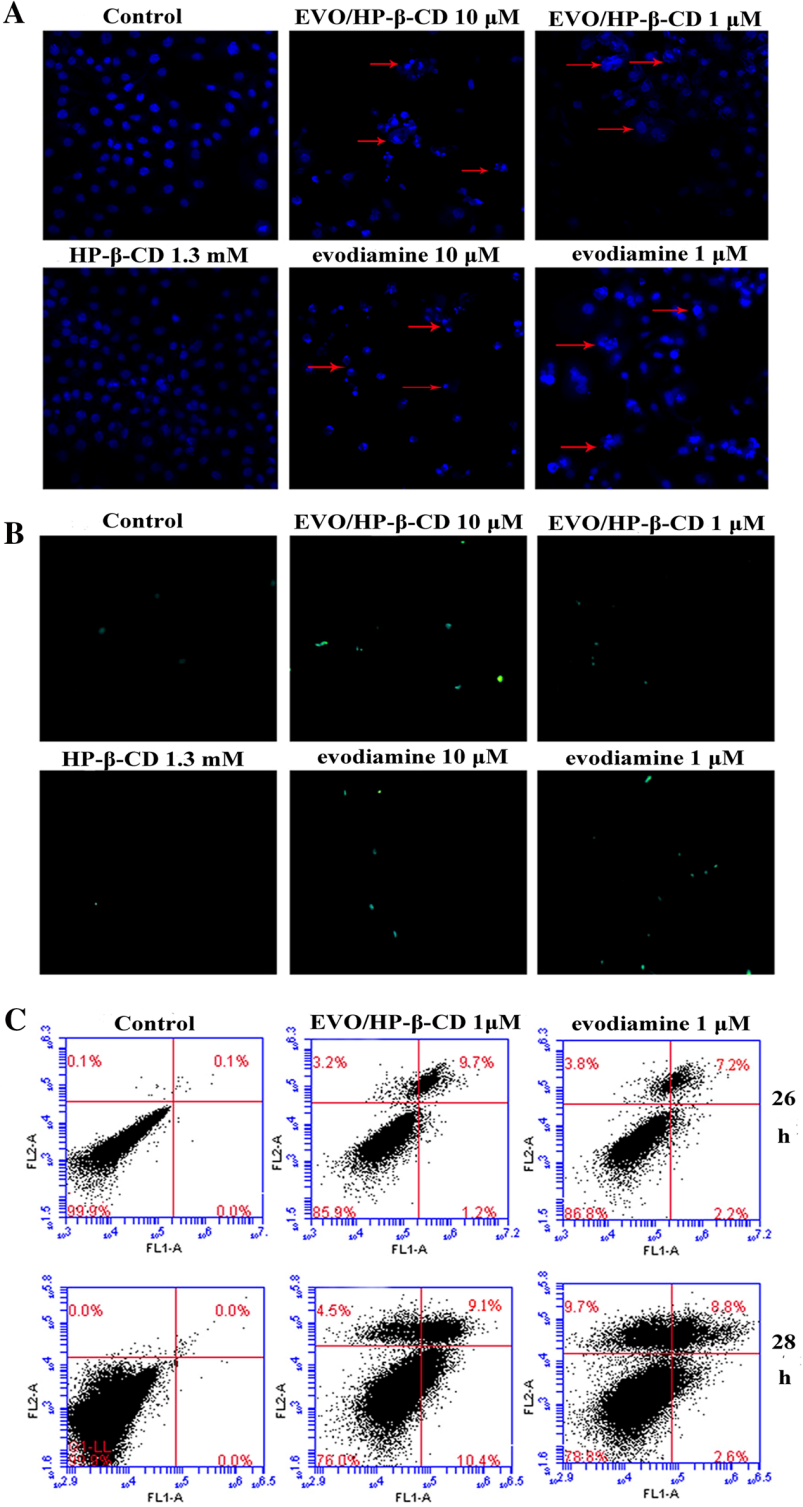
Study of apoptosis with staining. **A** Morphology of HepG2 cells after Hoechst staining (200× magnifications). **B** Apoptotic cells induced by evodiamine or EVO/HP-β-CD complexes after TUNEL assay (100× magnification). **C** Apoptosis induced by 1 μM evodiamine or EVO/HP-β-CD complexes for 26 or 28 h, respectively, and detected by flow cytometry analysis after staining with Annexin V-FITC/PI. The percentages in the *upper–right* and *lower–right* corners are the portions of late-stage and early-stage apoptosis, respectively

In general, apoptotic cells display some specific morphological and biochemical characteristics, including cell rounding and shrinkage, membrane blebbing, chromatin condensation [29], and mitochondrial inner membrane potential changes [30]. Hoechst 33258 is a cell-permeable nucleic acid stain that is very sensitive to DNA conformation and chromatin state. As shown in the photographs (Fig. 8A), morphological changes, such as chromatin condensation, were seen in the nuclei of the apoptotic cells induced by evodiamine and EVO/HP-β-CD. However, the karyomorphism of cells treated with HP-β-CD alone or with the vehicle was still very intact. The TUNEL assay was used to further confirm DNA fragmentation. As shown in Fig. 8B, positive signal was detected in HepG2 cells treated with evodiamine or EVO/HP-β-CD complexes. However, cells treated with HP-β-CD alone or with the vehicle do not exhibit cell-specific green fluorescence. These results showed that the apoptosis occurring in the HepG2 cells was induced by evodiamine or EVO/HP-β-CD complexes.

Flow cytometry combined with Annexin V-FITC/PI staining was also applied to confirm that the apoptosis occurring in the HepG2 cells was induced by evodiamine or EVO/HP-β-CD complexes. As shown in Fig. 8C, evodiamine and EVO/HP-β-CD complexes could trigger apoptosis in HepG2 cells in a time-dependent manner. Furthermore, EVO/HP-β-CD complexes could more effectively induce HepG2 cells apoptosis than evodiamine alone.

## Conclusions

The EVO/HP-β-CD inclusion complexes were prepared successfully. The DSC, FTIR, XRD, UV, ^1^HNMR and 2D ROESY results all suggest the formation of EVO/HP-β-CD complexes. Additionally, the antitumor activities of the EVO/HP-β-CD complexes on HepG2 cells were better than evodiamine alone, as confirmed by the MTT assay, caspase-3 assay, and two-colour analysis of FITC-AnnexinV/PI staining. This increase in antitumor activity can be attributed to the enhancement of evodiamine solubility and the inhibition of Pgp ATPase activity by HP-β-CD, which improved evodiamine cellular uptake. Based on our results, EVO/HP-β-CD complexes are very promising for drug delivery to enhance the bioavailability of evodiamine in vivo.

## Additional files

10.1186/s13065-016-0191-y ^1^HNMR chemical shift of evodiamine and its inclusion complex in DMSO-d_6_.
10.1186/s13065-016-0191-y A possible inclusion model for EVO/HP-β-CD inclusion complex.
