# Supplementary material for: A promising antitumor activity of evodiamine incorporated in hydroxypropyl-β-cyclodextrin: pro-apoptotic activity in human hepatoma HepG2 cells
Source: Chem Cent J. 2016 Jul 25;10:46. doi: 10.1186/s13065-016-0191-y (PMC4959055; doi:10.1186/s13065-016-0191-y)
Supplement: Supplementary file 1 — 10.1186/s13065-016-0191-y 1HNMR chemical shift of evodiamine and its inclusion complex in DMSO-d6. [file 13065_2016_191_MOESM1_ESM.docx]

Table S1. ^1^HNMR chemical shift of evodiamine and its inclusion complex in DMSO-d_6_

| evodiamine(H) | evodiamine(δ) | EVO/HP-β-CD(δ_1_) | Δδ |
| --- | --- | --- | --- |
| H-1 | 7.36 | 7.34 | 0.02 |
| H-2 | 6.96 | 6.94 | 0.02 |
| H-4 | 7.47 | 7.46 | 0.01 |
| H-5 | 11.07 | 11.05 | 0.02 |
| H-8 | 6.12 | 6.11 | 0.01 |
| H-10 | 7.79 | 7.78 | 0.01 |
| H-11 | 7.10 | 7.09 | 0.01 |
| H-12 | 7.00 | 6.99 | 0.01 |
| H-13 | 7.05 | 7.04 | 0.01 |
