# Supplementary figures and images for: A promising antitumor activity of evodiamine incorporated in hydroxypropyl-β-cyclodextrin: pro-apoptotic activity in human hepatoma HepG2 cells
Source: Chem Cent J. 2016 Jul 25;10:46. doi: 10.1186/s13065-016-0191-y (PMC4959055; doi:10.1186/s13065-016-0191-y)

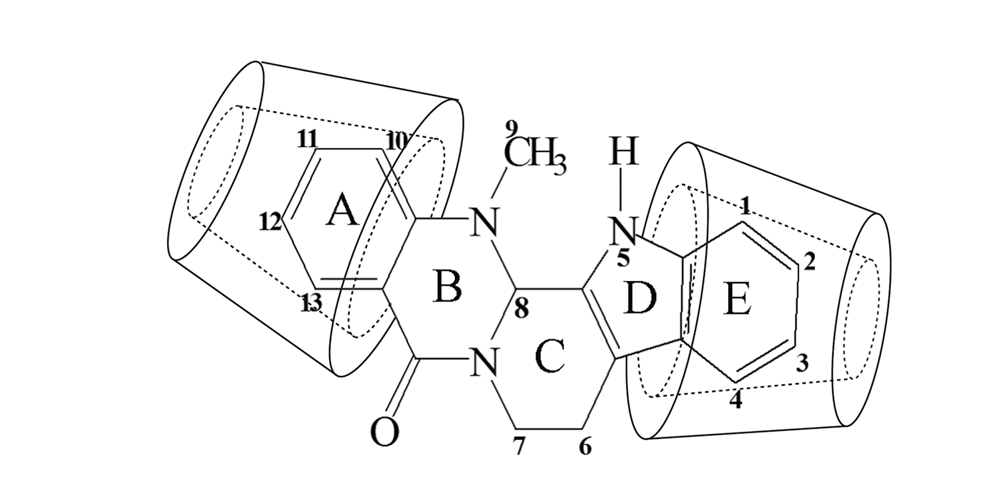

Supplement: Supplementary file 2 — 10.1186/s13065-016-0191-y A possible inclusion model for EVO/HP-β-CD inclusion complex. [file 13065_2016_191_MOESM2_ESM.tif]
